# Beyond Surgical Margins: Fully Mature Tertiary Lymphoid Structures (fmTLSs) Are Predictive Biomarkers for Local Recurrence in Primary Soft-Tissue Sarcomas

**DOI:** 10.3390/cancers18111685

**Published:** 2026-05-22

**Authors:** Audrey Michot, Lucile Vanhersecke, Derek Dinart, Aurélien Bourdon, Rihab Azmani, Valérie Velasco, Iris Bonomo, Maïlys Toureille, Maud Toulmonde, Raul E. Perret, Carine Bellera, Jean-Michel Coindre, François Le Loarer

**Affiliations:** 1 Bordeaux Institute of Oncology, BRIC U1312 Team 9 Sarcotarget, INSERM, Université de Bordeaux, 33000 Bordeaux, France; 2 Department of Surgery, Institut Bergonié, 33000 Bordeaux, France; bonomo.iris@gmail.com; 3 Department of Biopathology, Institut Bergonié, 33076 Bordeaux, France; l.vanhersecke@bordeaux.unicancer.fr (L.V.); v.velasco@bordeaux.unicancer.fr (V.V.); m.toureille@bordeaux.unicancer.fr (M.T.); j.coindre@bordeaux.unicancer.fr (J.-M.C.); 4 Clinical and Epidemiological Research Unit, INSERM CIC1401, Institut Bergonié, 33076 Bordeaux, France; d.dinart@bordeaux.unicancer.fr (D.D.);; 5 Department of Data and Digital Health, Institut Bergonié, 33076 Bordeaux, France; a.bourdon@bordeaux.unicancer.fr (A.B.); r.azmani@bordeaux.unicancer.fr (R.A.); 6 Oncologic Unit, Institut Bergonié, 33076 Bordeaux, France; m.toulmonde@bordeaux.unicancer.fr

**Keywords:** biomarker, Prognostic factor, tertiary lymphoid structures (TLS), tumor microenvironment, soft-tissue sarcoma (STS)

## Abstract

**Background:** Soft-tissue sarcomas (STSs) are rare and heterogeneous malignancies with generally poor and unpredictable prognosis. Tertiary lymphoid structures (TLSs) have been identified as favorable prognostic indicators in several cancer types, yet their role in STS remains poorly defined. This study investigates the prognostic relevance of TLS presence, maturity, location and density in resected STSs. **Methods:** We retrospectively analyzed 219 cases of primary STS surgically resected at the Bergonié Institute (France) between 1990 and 2020. TLSs were assessed for presence, spatial distribution, semi-quantitative density and degree of maturity using CD20 and CD23 immunohistochemistry, categorizing tumors as fully mature TLS-positive (fmTLS^+^) or -negative (fmTLS^−^). RNA sequencing was performed on 126 formalin-fixed paraffin-embedded samples to characterize immune microenvironment profiles. Survival outcomes—including overall survival (OS), time to locoregional progression (TTLRP), and time to distant progression (TTDP)—were analyzed using Kaplan–Meier estimates and Cox proportional hazards models. **Results:** The presence of fmTLS was significantly associated with improved 5-year OS (*p* = 0.012) and cause-specific survival (*p* = 0.006). Unexpectedly, fmTLS^+^ tumors showed a higher rate of local recurrence (22.9% vs. 8.1%, *p* = 0.002). On multivariate analysis, high-density fmTLS^+^ tumors conferred a 2.68-fold increased risk of locoregional progression (95% CI: 1.28–5.59, *p* = 0.009). Transcriptomic profiling confirmed a significant correlation between fmTLS^+^ status and a high-immune phenotype (Φ = 0.30, *p* < 0.001). **Conclusions:** STSs with fmTLS are associated with improved OS but increased risk of local recurrence. These findings support fmTLS as a dual prognostic biomarker and highlight the need for tailored surveillance and adjuvant strategies in fmTLS^+^ patients.

## Simple Summary

This study establishes fully mature tertiary lymphoid structures (fmTLSs) as a dual prognostic biomarker in primary soft-tissue sarcomas (STSs) of the limbs and trunk wall. By systematically analyzing 219 resected STS specimens, we show that fmTLS presence correlates with improved 5-year overall survival, independent of conventional histopathological factors, yet unexpectedly predicts a significantly higher risk of local recurrence after surgery. These findings suggest that fmTLS may identify patients with robust systemic anti-tumor immunity but who remain at risk for locoregional relapse, potentially due to residual microscopic disease. Incorporating fmTLS assessment into routine pathological evaluation could refine current risk-stratification algorithms, complement existing grading systems and inform personalized surveillance or adjuvant therapy decisions. Future integration of immune microenvironment profiling with molecular and clinical data may further individualize sarcoma management and improve patient outcomes.

## 1. Introduction

Soft-tissue sarcomas (STSs) are a heterogeneous group of malignant mesenchymal tumors, characterized by diverse histological patterns and highly variable clinical behavior. Among them, pleomorphic sarcomas are the most frequent subtype in patients over 50 years of age and are typically associated with complex genetic alterations and poor outcomes, often requiring extensive surgical resections [1].

Prognosis in localized STS currently relies mainly on histological grading, with the French *Fédération Nationale des Centres de Lutte Contre le Cancer* (FNCLCC) system being the most widely used, together with tumor size, depth and anatomical site (limbs vs. trunk wall) [2,3,4,5]. Several nomograms, such as Sarculator, integrate these variables and histological subtype to refine individual risk estimates but substantial uncertainty remains for many patients [3,6,7,8].

For localized STS of the limbs and trunk wall, wide *en-bloc* surgical excision remains the standard of care [9]. However, despite complete resection (R0), 40–50% of patients experience local recurrence or metastasis within 5 years [2,10,11]. While adjuvant chemotherapy may benefit high-risk patients [12], and radiotherapy improves local control, the risk of recurrence remains substantial [13,14]. Re-excisions for local recurrence, especially after radiotherapy, are often associated with high morbidity, highlighting the urgent need for robust prognostic biomarkers to better identify patients at risk and guide personalized treatment strategies. In advanced disease, targeted therapies and immune checkpoint inhibitors have expanded the therapeutic landscape for specific molecular subsets of STS, but their benefit remains limited and resistance is frequent [15,16,17]. Recent work also suggests that chromatin organization and epigenetic dysregulation contribute to shaping transcriptional programs and immune-related states, potentially influencing sarcoma behavior and response to modern therapies [18,19].

Recent studies have underscored the critical role of the tumor immune microenvironment in STS progression and therapeutic response. Among immune features, tertiary lymphoid structures (TLSs)—organized aggregates of B cells resembling lymph nodes [15,20,21,22]—have emerged as strong predictors of survival in various cancer types, including sarcomas [16,17,23,24,25,26,27,28,29,30,31,32,33,34,35,36]. Mature TLS (mTLSs), defined as aggregates of B lymphocytes containing CD21+CD23+ follicular dendritic cells (FDCs) [37], appear particularly relevant, outperforming conventional markers such as CD8+ T cell density and PD-L1 expression [37]. In advanced STS, TLSs have also been proposed as predictive biomarker for response to ICI [38].

Despite these promising insights, the prevalence and spatial distribution of TLS in sarcomas remain poorly characterized. Most available data are derived from small biopsy samples, limiting comprehensive spatial analysis. Moreover, as anti-tumor immune responses predominantly occur at the invasive front [39], biopsy-based assessments may not accurately capture TLS heterogeneity compared with whole resection specimens. In addition, TLS status is often evaluated in metastatic rather than primary tumors. TLS maturation also exists along a continuum, from loose lymphoid aggregates to fully organized structures with germinal centers and follicular dendritic cells: fully mature TLSs (fmTLSs) with germinal centers and/or CD23+ FDC meshworks may represent the most functionally relevant stage [28,29,37,38]. However, in primary localized STS, the prevalence, spatial distribution, density and prognostic impact of fmTLS within whole surgical specimens remain largely undefined.

Taken together, these observations highlight a major gap: the lack of robust immune-based prognostic biomarkers in localized STS derived from systematic evaluation of whole resection specimens and accounting for TLS maturity, density and spatial distribution.

We therefore hypothesized that the presence, maturity and density of TLS, and in particular fully mature TLS (fmTLS), in primary STS of the limbs and trunk wall could serve as clinically relevant prognostic biomarkers beyond conventional histopathological factors. To test this hypothesis, we conducted a systematic analysis of TLS in a large cohort of 219 surgically resected primary STS specimens and investigated their association with clinical outcomes, focusing on overall survival, locoregional control and transcriptomic immune profiles.

## 2. Materials and Methods

### 2.1. Patient Selection

This retrospective study included data from the national clinico-biological sarcoma database (SarcomaBCB, https://conticabase.sarcomabcb.org). Eligible patients were adults (aged ≥ 18 years) who underwent surgical resection for a primary soft-tissue sarcoma of the limb or the trunk wall at the Institut Bergonié (Comprehensive Cancer Center, Bordeaux, France) between January 1990 and December 2020. Histological diagnosis was confirmed by expert pathologists within the French national sarcoma network (RRePS/NetSarc). Patients with metastatic disease at diagnosis or who received neoadjuvant treatments were excluded. The study protocol was approved by the institutional review board and conducted in compliance with the Declaration of Helsinki. All data were recorded within the national NETSARC database, approved by the French data protection authority (CNIL, no. 910390). The requirement for informed consent was waived due to the retrospective nature of the study and the use of archived diagnostic specimens, in accordance with French legislation.

We further acknowledge that the monocentric design may limit the generalizability of our findings and that external validation in independent cohorts will be required.

### 2.2. Sample Collection

A total of 219 surgical specimens were retrieved from the Pathology department archives of the Institut Bergonié. All available formalin-fixed paraffin-embedded (FFPE) blocks for each case were reviewed. Representative blocks were selected to include the invasive front and the surrounding tissue, prioritizing areas most likely to harbor lymphoid aggregates and TLS. On average, 12 blocks per patient were evaluated (range 1–46), allowing a comprehensive assessment of TLS distribution within the whole resection specimen.

### 2.3. Assessment of Tertiary Lymphoid Structure (TLS) Status

**Figure 1:**
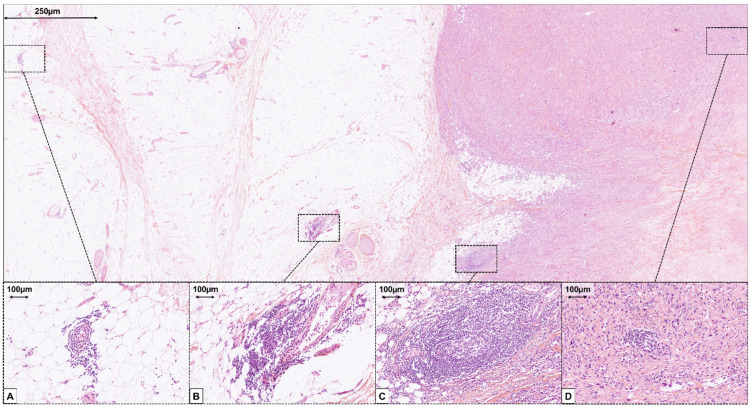
Distribution of TLS in the different areas of the tumors. This is a representative example of an undifferentiated pleomorphic sarcoma (UPS) showing TLS categorized according to their location to the tumor. (**A**) TLS located in non-tumoral tissue beyond 1 cm of the tumor were classified “NT”. (**B**) TLS were classified in “R1” zone when present beyond the tumor invasion front, up to 1 cm from the tumor. (**C**) TLS at the periphery of the tumor, i.e., in contact with the tumor invasion front and with non-tumoral area, were labeled “PT”. (**D**) TLSs within the tumor bulk (intermingled with tumor cells) were categorized as “CT”. All histology slides were stained with hematoxylin–eosin–saffron (HES). The scale bars indicate 250 µm on the upper picture, and 100 µm on all lower pictures.

All hematoxylin eosin and saffron (HES) slides were reviewed by an expert pathologist (L.V.) to assess TLS, using previously validated morphological criteria [39]. Lymphoid aggregates containing ≥ 50 mononuclear cells were identified as candidate TLSs. The TLS evaluation workflow is illustrated in Supplementary Figure S1. Each sample was assessed for (1) TLS presence on HES, scored as 0 (absent), 1 (indeterminate, requiring IHC), or 2 (mature TLS [mTLS] with visible germinal centers (GC)) (Supplementary Figure S2), (2) TLS spatial distribution categorized as CT (tumor center), PT (periphery), R1 (reactive zone < 10 mm from tumor margin without direct contact), or NT (normal tissue > 10 mm) (Figure 1) and (3) a semi-quantitative TLS density score based on the average number of lymphoid aggregates per slide, calculated across all slides analyzed for each case, defined as 1+ (<1 aggregate/slide), 2+ (1–5 aggregates/slide), 3+ (>5 aggregates/slide) (Supplementary Figure S3).

**Figure 2:**
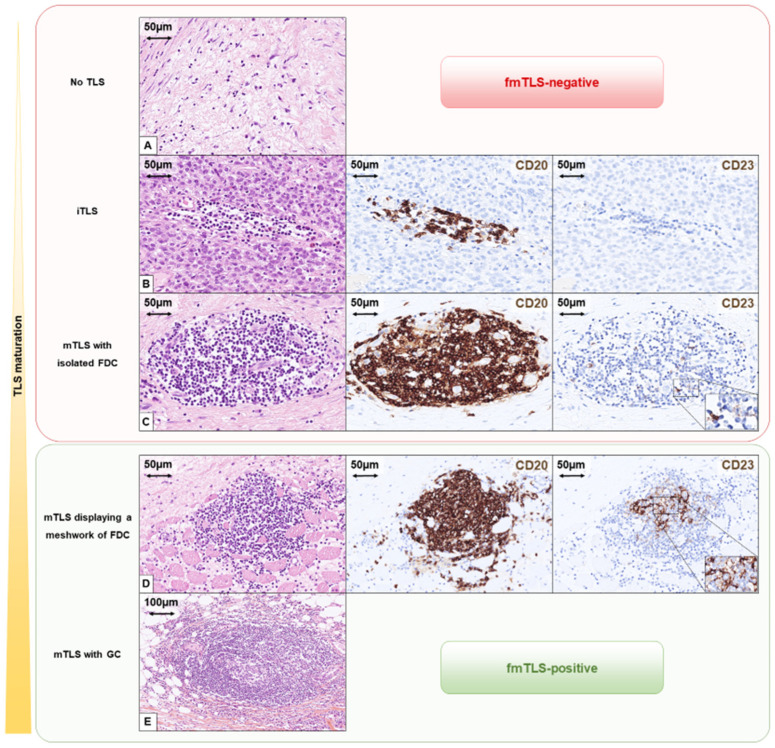
Classification of tertiary lymphoid structures (TLS). Samples were separated in two main groups: “fully mature TLS-negative (fmTLS^−^)” gathered (**A**) specimens without TLS, (**B**) immature TLS (iTLS) without CD23+ follicular dendritic cells (FDC), and (**C**) mature TLS (mTLS) with only scattered CD23+ FDC. “Fully mature TLS-positive (fmTLS^+^)” brought together (**D**) mTLS displaying a meshwork of CD23+ FDC and (**E**) mTLS with germinal center visible on HES staining. All histology slides were stained with hematoxylin–eosin–saffron (HES). The stainings are indicated on the upper part of the image. The scale bars indicate 50 µm in size for pictures (**A**–**D**), and 100 µm for picture (**E**). Slides (**A**,**B**) were issued from solitary fibrous tumors. Slide (**C**) came from a myxofibrosarcoma. Slides (**D**,**E**) were found in undifferentiated pleomorphic sarcomas.

For indeterminate TLS (score 1), CD20 and CD23 immunohistochemistry (IHC) tests were performed on serial FFPE sections [25]. TLSs were categorized as immature TLS (iTLS), partially mature (mTLS with isolated CD23+ FDC), and fully mature (mTLS with FDC meshwork of CD23+ defined as ≥4 interconnected CD23+ FDCs forming a network (Figure 2). Samples were grouped as fully mature TLS-negative (fmTLS^−^) if TLSs were absent or immature or displayed only isolated FDCs, and fully mature TLS-positive (fmTLS^+^) if they showed HES-visible GC or FDC meshworks (Figure 2 and Supplementary Figure S1). All TLS assessments were performed by a single expert pathologist. Interobserver reproducibility was not evaluated in this study and is therefore acknowledged as a limitation for the routine implementation of fmTLS scoring.

### 2.4. Immunohistochemistry

IHC was performed on 3 µm FFPE sections using the Ventana Benchmark Ultra platform (Ventana Medical Systems, Tucson, AZ, USA). Detection was carried out with ultraView DAB or OptiView DAB kits (Roche Diagnostics, Tucson, AZ, USA). CD20 (clone L26, prediluted, Roche/Ventana, Tucson, AZ, USA) and CD23 (clone SP23, prediluted, Roche/Ventana, Tucson, AZ, USA) antibodies were used following standardized protocols (Cell Conditioning 1 for 64 min; antibody incubation 36 min). Detailed conditions are provided in Supplementary Table S1.

### 2.5. Statistical Analysis

Descriptive statistics included means and standard deviations (SD) for continuous variables and proportions for categorical variables. Group comparisons were performed using Fisher’s exact test or Chi-squared tests as appropriate. Overall survival (OS) was defined from date of diagnosis to death from any cause. Time to distant progression (TTDP) and time to locoregional progression (TTLRP) were defined from the date of diagnosis to the date of first distant or locoregional event (local recurrence or regional nodal progression), respectively, and were censored at last follow-up or death. TTLRP thus reflects the time to the first locoregional event, regardless of whether it is local or regional. Kaplan–Meier curves and log-rank tests were used for univariate survival analyses. Prognostic factors were assessed using multivariate Cox proportional hazards models, employing backward selection with *p* < 0.05. Covariates included age, gender, tumor size (<5 cm vs. 5–10 cm vs. >10 cm), site and depth of tumor, locoregional extension (vascular, nervous or bone invasion), FNCLCC histological grade, multifocality, histologic subtype, surgical margins, surrounding tissue, adjuvant therapy, molecular class of sarcoma, anatomical tumor limitation, fmTLS status and fmTLS semi-quantitative density score. TLS–immune correlation was evaluated using the Phi coefficient and immune classification agreement was estimated using Gwet’s AC1 coefficient. We did not perform competing-risk analyses, and death was treated as a censoring event in the TTDP and TTLRP analyses.

### 2.6. Transcriptomic Analysis and Immune Profiling

RNA was extracted from FFPE samples available from 2008 to 2020 (66.7%, n = 146/219). Earlier specimens (1992–2008) fixed in Bouin Hollande’s solution were unsuitable for RNA extraction. For each sample, four regions (CT, PT, R1, NT) were microdissected separately. RNA libraries were prepared using the TruSeq RNA Access Kit (Illumina, San Diego, CA, USA) and sequenced on a NextSeq 500 platform (Illumina, San Diego, CA, USA) [40]. Twenty samples were excluded due to quality control failure, leaving 126 high-quality transcriptomes (86.3% of the initially extracted samples, N = 126/146).

Gene expression quantification was performed using HTSeq v0.6.0 [41] and normalized using the VOOM method. Unsupervised clustering was conducted using hierarchical clustering (1-Pearson_correlation, hclust function in R version 4.3.1; R Foundation for Statistical Computing, Vienna, Austria) [42]. Consensus clustering was performed with the package ConsensusClusteringPlus package version 1.62.0 from BioConductor [43].

Immune subgroups were categorized into immune-high (group E), immune-low (group A), or non-A/non-E (intermediate) groups based on a modified version of Petitprez et al.’s immune classification [16]. Following the approach of Petitprez et al., the final immune status dichotomization (immune-high vs. non-immune-high) focused on the tumor and the margin, excluding distant healthy tissue to maintain focus on the immediate tumor microenvironment. For downstream prognostic analyses, the transcriptomic immune status was therefore considered as a binary variable (immune-high vs. non-immune-high).

## 3. Results

### 3.1. Cohort Characteristics

We analyzed 219 primary soft-tissue sarcoma (STS) surgical specimens from patients treated between 1990 and 2020. The clinicopathological characteristics are summarized in Supplementary Table S2. The majority of tumors were deep-seated tumors (90.0%, N = 197/219), with 24.2% (N = 53/219) extending into the superficial soft tissues. Most tumors were larger than 5 cm (82.2%, N = 180/219, size range 13–310 mm). FNCLCC grade 3 tumors accounted for 45.7% of cases (N = 100/219) (Supplementary Table S2). Of note, in this study, every case was graded according to the criteria reported in Coindre JM et al. [5]. The median follow-up was 102.3 months. During this period, 32 patients (N = 32/219, 14.6%) experienced at least one local recurrence, 72 patients (N = 72/219, 32.9%) developed metastases and 86 patients (N = 86/219, 39.3%) died of disease (Supplementary Table S2). Among the 32 patients with local relapse, 24 (10.9% of the whole cohort) presented local recurrence as the first event. These 24 events, together with 9 isolated regional nodal progressions, contributed to the 33 locoregional events considered in the TTLRP analyses.

### 3.2. TLS Prevalence and Maturity

Overall, lymphoid aggregates considered as potential TLS on HES-stained slides were identified in 83.6% (N = 183/219) of patients. Among these, TLSs were confirmed in 90.1% of cases (N = 166/183), highlighting the need for CD20 immunohistochemistry to ascertain the presence of B cells in structures lacking a germinal center. TLS^+^ samples represented 75.8% of the entire cohort (N = 166/219), and fully mature TLSs (fmTLSs) were observed in 57.8% of them (N = 96/166), corresponding to 43.8% fmTLS^+^ patients in the whole cohort (N = 96/219) (Table 1, Supplementary Figure S1). Interestingly, only 41.7% (N = 40/96) of fmTLS displayed visible GC on HES slides, emphasizing the importance of CD23 immunohistochemistry for accurate classification (Supplementary Table S3).

### 3.3. Lymphoid Aggregates Distribution and Density

Lymphoid aggregates were heterogeneously distributed across tumor compartments. Among 2575 tumor blocks reviewed (mean: 12 blocks per patient, range: 1–46), the R1 zone (tumor front margin) was the most frequent site of TLS detection, with 90.2% (N = 165/183) of lymphoid aggregates-positive cases having at least 1 aggregate in this area, and 56.3% (N = 103/183) of them having lymphoid aggregates preferentially located in the R1 area (Supplementary Table S4). Lymphoid aggregates were also observed in the tumor periphery (PT) and tumor center (CT), but to a lesser extent, being preferentially located in the PT in 14.2% of cases (N = 26/183) and in the CT in 27.9% of cases (N = 51/183). Lymphoid aggregates confined predominantly to non-tumoral distant tissue (NT) were rare, occurring in only 1.6% of patients (N = 3/219). The R1 zone remained the predominant location regardless of TLS maturity (Supplementary Table S5). Lymphoid aggregates density, assessed semi-quantitatively (scores: 1+, 2+, 3+) was evenly distributed across samples (Supplementary Table S4). High density lymphoid aggregates (scores 2+/3+) were strongly associated with fmTLS maturity: 85.4% (N = 82/96) of fmTLS^+^ samples were associated with high density (2+/3+) scores (Supplementary Table S5). Reciprocally, 69.5% (N = 82/118) of samples with high density (2+/3+) scores were fmTLS^+^. In contrast, 78.5% (N = 51/65) of low-density lymphoid aggregates samples (score 1+) were fmTLS^−^ (Supplementary Table S5), highlighting a positive correlation between lymphoid aggregates density and maturity.

### 3.4. TLS Prevalence Across Sarcoma Subtypes

FmTLS were most prevalent in dedifferentiated liposarcomas (DDLPS, 63.2%, N = 12/19) and undifferentiated pleomorphic sarcomas (UPS, 56.6%, N = 30/53). Conversely, myxoid/round cell liposarcomas showed the lowest prevalence (MRCLPS, 25.0%, N = 7/28) (Table 1).

Sarcomas with complex genetic alterations harbored fmTLS more frequently than those with simple genomics (76.0% vs. 60.2%, respectively, *p* = 0.013). Likewise, fmTLS increased with higher FNCLCC (Table 1).

### 3.5. Prognostic Impact of TLS

**Figure 3:**
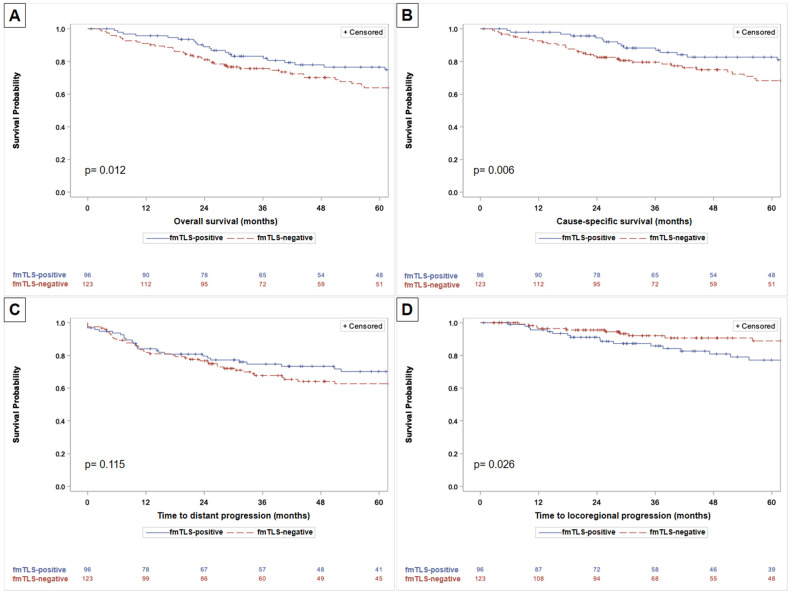
Impact of fmTLS on outcome in patients with primary resected sarcomas: (**A**) 5-year overall survival (OS) for fmTLS^+^ and fmTLS^−^ populations; (**B**) 5-year cause-specific survival for fmTLS^+^ and fmTLS^−^ populations; (**C**) 5-year time to distant progression (TTDP) for fmTLS^+^ and fmTLS^−^ populations; (**D**) 5-year time to locoregional progression (TTLRP) for fmTLS^+^ and fmTLS^−^ populations.

Prognostic factors significantly associated with 15-years OS were age, tumor size, locoregional extension, histotype, genomic molecular class, FNCLCC histological grade, quality of surgical resection and adjuvant chemotherapy (Table 2). Of note, fmTLS status did not significantly impact on 15-years OS (*p* = 0.466), nor did the presence of mature TLS (mTLS, *p* = 0.651). Multivariate analysis revealed that age, locoregional extension, histotype, quality of resection and anatomical tumor limitation were independently associated with 15-years OS (Table 2). Regarding the 5-years survival, fmTLS^+^ status was significantly associated with better OS (*p* = 0.012) (Figure 3A) and cause-specific survival (*p* = 0.006) (Figure 3B) compared to fmTLS^−^ samples. These findings are consistent with a predominant short- to mid-term effect of fmTLS on survival, which attenuates over longer follow-up.

For distant progression, 15-years TTDP was significantly affected by age, tumor size, genomic molecular class, FNCLCC grade and surgical margins in univariate analysis (Supplementary Table S6). In multivariate analysis, only age, tumor size and histotype remained independent predictors (Supplementary Table S6). While fmTLS^+^ sarcomas showed a favorable trend towards prolonged time to distant progression (TTDP) at 5-years, this did not reach statistical significance (*p* = 0.115) (Figure 3C). Of note, TTDP did not differ significantly based on mTLS status (*p* = 0.864).

Unexpectedly, when considering any local recurrence during follow-up, fmTLS^+^ tumors had a significantly higher rate of local recurrence compared to fmTLS^−^ tumors (22.9% vs. 8.1% respectively, *p* = 0.002) (Table 1). In the TTLRP analyses, which considered time to first locoregional event (local recurrence as first event or regional nodal progression), 33 events were observed. TTLRP was indeed significantly impacted by age, genomic molecular class, FNCLCC grade, surgical margins, adjuvant radiotherapy and fmTLS status (Table 3, Figure 3D). The association became even stronger when combining the fmTLS status and the TLS density. Indeed, multivariate analysis demonstrated that only advanced age and high density fmTLS^+^ (score 2+/3+) were significantly associated with shorter TTLRP (Table 3). Patients with high density fmTLS^+^ had a 2.68-fold increased risk of locoregional progression compared to those with low-density or fmTLS^−^ tumors (95% CI = 1.28–5.59, *p* = 0.009) (Table 3). Again, TTLRP did not differ significantly based on the mTLS status (*p* = 0.852).

### 3.6. Transcriptomic Immune Profiling

Transcriptomic analysis revealed that 33.3% (N = 42/126) samples were classified as immune-high (Supplementary Table S7). Immune-low and intermediate cases represented 38.1% (N = 48/126) and 28.6% (N = 36/126), respectively.

The transcriptomic immune status did not significantly correlate with overall survival (OS), time to distant progression (TTDP), or time to locoregional progression (TTLRP) (Supplementary Table S8).

Then, we compared transcriptomic immune classification with the histologically defined fmTLS status in the 126 cases for which both data types were available. While fmTLSs were present in 45.2% samples (N = 57/126), only 33.3% were immune-high by transcriptomic analysis, suggesting that pathological detection may be more sensitive for capturing localized immune structures (Supplementary Table S9). The concordance between the two metrics was moderate but significant (Phi coefficient = 0.30, *p* value <0.001) (Supplementary Table S9).

These results indicate that although the fmTLS and transcriptomic immune classifications are not interchangeable, they are positively correlated and reflect related aspects of the tumor immune microenvironment.

## 4. Discussion

By analyzing a large and well-annotated cohort of 219 surgically resected primary STS of the limbs and trunk wall, we investigated the immune landscape—focusing on TLS—through a combined approach of comprehensive pathological screening and transcriptomic analysis.

### 4.1. TLS Heterogeneity Within and Across STS Subtypes

Our systematic comprehensive pathological assessment revealed TLS in 75.8% of STS, a prevalence higher than previously reported [32]. This likely reflects our exhaustive evaluation of entire surgical specimens and the targeted selection of FFPE blocks most likely to contain TLS, in contrast to studies relying on unselected blocks or core-needle biopsies that may underestimate TLS presence [39].

Spatial analysis showed that TLS could be detected across all tumor compartments, although distribution was heterogeneous. The R1 zone was the predominant location, with 90.2% of lymphoid aggregate-positive cases harboring aggregates in this area. Due to this spatial heterogeneity and the simultaneous presence of TLS at various maturation stages, we could not independently evaluate the clinical significance of each maturation stage per region. To better account for this distribution complexity, we employed a global semi-quantitative score based on the entire specimen to better capture the true distribution within the whole sample.

We observed a strong positive correlation between lymphoid aggregate density and TLS maturity: tumors with higher density were more likely to harbor fmTLS. Notably, fmTLS prevalence also varied across sarcoma subtypes, being highest in dedifferentiated liposarcomas and undifferentiated pleomorphic sarcomas and lowest in myxoid/round cell liposarcomas. This difference highlights substantial inter- and intra-tumoral heterogeneity in immune responses and suggests that immune surveillance and evasion mechanisms may differ by histotype.

### 4.2. Dual Impact of fmTLS

The presence of fmTLS was significantly associated with improved 5-year OS as well as cause-specific survival, consistent with previous studies reporting the positive prognostic impact of TLS in various malignancies [15,24,27,28]. Similarly, a trend toward longer 5-year time to distant progression (TTDP) was observed but did not reach statistical significance.

Importantly, our study is the first to investigate the impact of TLS maturity and density on local recurrence in surgically treated STS. Unexpectedly, we found that a high density of fmTLS (i.e., ≥1 TLS per slide on average) was significantly associated with a 2.68-fold increased risk of local recurrence, regardless of TLS location within the sample. This effect was independent of TLS location and persisted in multivariate analysis, suggesting that fmTLS may act as microscopic sentinels, identifying patients at elevated risk for local recurrence post-surgery, independent of surgical margin status.

The duality—fmTLS correlating with both better survival and higher local recurrence—highlights the complex and nuanced nature of immune–tumor interactions in STS. While the presence of fmTLS appears to support systemic anti-tumor immunity, it may also indicate localized immune activation that fails to fully eradicate residual tumor progenitor cells. These interpretations remain hypothetical and represent possible explanation that will require dedicated functional and spatial studies. Similar mechanisms have been proposed in hepatocellular carcinoma [44].

Other studies have highlighted the complex interplay between immune factors and sarcoma outcomes. Smolle et al. reported significantly increased expression of immune checkpoint markers (PD-L1, PD-1) and tumor-infiltrating lymphocyte (TIL) subsets in myxofibrosarcomas compared to others [45]. Notably, myxofibrosarcomas are characterized by a high rate of local recurrence [46]. Moreover, the presence of regulatory T cells (Tregs) was significantly associated with increased local recurrence risk, independent of margins [45]. While TLS status was not assessed in the study, these findings suggest that multiple immunological parameters may contribute to identifying patients at high risk of recurrence.

To advance understanding of the paradoxical association of fmTLS with both improved survival and increased local recurrence, future studies should focus on the immune cell composition and functional states within TLS in recurrent versus non-recurrent tumors. Comprehensive approaches such as multiplex immunohistofluorescence and spatial transcriptomic profiling can provide high-resolution characterization of immune subsets, including Tregs, exhausted CD8+ T cells, and macrophages within and around TLS. Recent evidence suggests that exhausted CD8+ T cells, characterized by high expression of inhibitory receptors (e.g., PD-1, LAG3, CTLA4) and diminished effector function, accumulate in the tumor microenvironment and within TLS, where they may reflect ongoing but ineffective anti-tumor response [47].

Other studies could investigate the spatial distribution and dynamic evolution of TLS throughout tumor progression—from diagnosis to relapse and metastasis—and under the influence of treatment. Such studies could provide critical insights into how TLS contribute to tumor–immune interactions over time and under therapeutic pressure. In addition, recent work on chromatin organization and epigenetic dysregulation has highlighted how epigenetic mechanisms can shape transcriptional programs and immune-related states in cancer, potentially interacting with TLS-associated immune phenotypes [18].

### 4.3. Clinical Practice Implications

The apparent contradiction in the clinical relationship between fmTLS and patient outcomes aligns with the understanding that local recurrence, while necessitating debilitating secondary surgeries, does not always significantly impact overall prognosis [13]. Our findings highlight the need to refine treatment strategies for patients with localized, non-metastatic sarcoma of the limbs or the trunk walls, to prevent both local recurrences and metastasis following surgical resection [9]. In this context, fmTLS assessment may complement FNCLCC grading and other standard prognostic parameters, offering a simple, reproducible tool to identify patients who may benefit from tailored surveillance or adjuvant strategies. However, any potential use of fmTLS in routine practice will require external validation, assessment of interobserver reproducibility, and evaluation of feasibility in non-specialized pathology settings, even though previous work by Vanhersecke et al. has already demonstrated the feasibility in other tumor types [39].

### 4.4. Design of the Study

The strength of this study lies in its meticulous design, incorporating long-term follow-up data and homogeneous therapeutic management, enabling robust comparative analyses. Moreover, this large retrospective study reflects the real-life experience of most sarcoma treatment centers. While our cohort size is substantial for STS research, larger and multi-center studies would be beneficial to validate our findings across diverse patient populations. However, it is worth noting that the importance of multicenter studies in STS is somewhat mitigated in this case, as our study was conducted in a reference Comprehensive Cancer Center. In accordance with the French National Cancer Institute (INCa) guidelines, patients from the entire *Nouvelle-Aquitaine* region are referred to our center, reducing the risk of a monocentric bias.

The significant prognostic impact of fmTLS at 5-year overall survival, despite the lack of statistical significance at 15 years, highlights the relevance of short- to mid-term prognostic markers in STS.

This finding is particularly relevant in the context of soft-tissue sarcomas, which are generally characterized by poor prognosis. We also acknowledge that the attenuation of the fmTLS effect over longer follow-up raises the possibility of time-dependent hazards; although the proportional hazard assumption was checked in our models, more refined time-dependent analyses would be of interest in future studies.

Because our work focused on resected specimens, future studies should investigate TLS status in unresectable or metastatic STS and explore longitudinal changes in TLS status at diagnosis, relapse and metastasis. Finally, TTDP and TTLRP were analyzed using standard Kaplan–Meier and Cox models, with death treated as a censoring event rather than a competing event; competing-risk approaches might provide more accurate estimates of cumulative incidence and represent an additional methodological refinement for future work.

### 4.5. Pathological Method of TLS Screening

While the definition of TLS remains highly heterogeneous and under refinement, we adopted validated criteria for routine pathology practice [37,39]. In our study, both CD20 and CD23 immunohistochemistries proved essential for accurate TLS identification and maturation assessment: among 83.6% of patients with lymphoid aggregates on HES slides, only 75.8% were confirmed as TLS by CD20 staining, and less than half (41.7%) of fmTLS exhibited visible germinal centers on HES, reinforcing the need for CD23 staining to detect the follicular dendritic cell network.

It remains important to recognize that discrepancies in TLS assessment criteria across studies are primarily due to the continuum of TLS maturation rather than a set of clearly distinct categories. We grouped all maturation stages into two categories: “fully mature TLS-positive” (fmTLS^+^) and “fully mature TLS-negative” (fmTLS^−^). Only the most mature stage—fmTLS, defined by germinal centers visible on HES and/or a distinct CD23+ follicular dendritic cell network—demonstrated prognostic relevance. Notably, mature TLS (mTLS) with only isolated CD23+ dendritic cells (fmTLS^−^) did not significantly impact OS, TTDP, or TTLRP, suggesting they are not functionally equivalent to fmTLS. We acknowledge that this binary classification may oversimplify a biological continuum, and that more granular or continuous metrics of TLS maturation and density might capture additional prognostic information.

### 4.6. Transcriptomic Analysis and Immune Profiling

Interestingly, immune status results based on transcriptomic analysis were concordant with the pathological TLS status. Approximately two-thirds of samples were “not immune high”, consistent with the immunogenically “cold” profile of sarcomas, with a low level of immune infiltration [18]. Our study demonstrated the heterogeneity of immune transcriptomic status between different tumor areas. When directly comparing TLS status with the corresponding transcriptomic profile of the specimen, we observed a moderate but significant agreement (Phi coefficient = 0.30). This suggests that transcriptomics, although based on a single sample, can sensitively capture immune-related features—possibly due to the detection of immune pathway activation, including B cell signaling, even in the absence of fully formed TLS in the sampled area. Conversely, histological TLS assessment provides spatially resolved information on localized immune aggregates that may not be fully captured by bulk RNA sequencing. Together, these complementary approaches highlight distinct but related facets of the tumor immune microenvironment.

## 5. Conclusions

In conclusion, this study highlights the dual role of fmTLS as a promising but complex prognostic biomarker in resected STS of the limbs and trunk wall. The presence of fmTLS correlated with improved 5-year OS, independently of conventional factors such as the FNCLCC grade, while being associated with a higher risk of local recurrence, regardless of margin status. This suggests that fmTLSs may serve as sentinels of microscopic residual disease, although this hypothesis requires further mechanistic validation.

Given the high prevalence of TLS when the entire surgical specimens are examined, a key priority for future research is to better account for TLS density—rather than relying on a binary classification—and to refine TLS maturation classification within a more nuanced, continuum-based framework. Additional studies are needed to clarify whether TLSs actively contribute to local recurrence or predominantly reflect an underlying inflammatory and/or immune state. Ultimately, integrating immune microenvironment features with histologic, molecular input and clinical parameters may support more personalized prognostic and therapeutic strategies in sarcomas, traditionally considered immunologically “cold”.

## Figures and Tables

**Table 1:** Comparison of clinical variables of patients with fmTLS^+^ versus fmTLS^−^ tumors.

	fmTLS^−^ (N = 123)		fmTLS^+^ (N = 96)		*p* Value (Chi-2)
	N	%	N	%	
**Sex**					0.7278
Female	58	47.2	43	44.8	
Male	65	52.8	53	55.2	
**Age at diagnosis (years)**					0.2788
<65	68	55.3	46	47.9	
≥65	55	44.7	50	52.1	
**Tumor site**					**0.0351**
Lower limb	85	69.1	52	54.2	
Trunk wall	29	23.6	28	29.2	
Upper limb	9	7.3	16	16.7	
**Tumor depth**					**0.0144**
Deep	91	74.0	53	55.2	
Superficial	9	7.3	13	13.5	
Superficial and deep	23	18.7	30	31.3	
**Tumor size**					0.4891
<5 cm	21	17.1	18	18.8	
5 to 10 cm	54	43.9	48	50.0	
>10 cm	48	39.0	30	31.3	
**Multifocality**					0.6307
No	109	88.6	87	90.6	
Yes	14	11.4	9	9.4	
Locoregional extension *					0.9243
No	111	90.2	87	90.6	
Yes	12	9.8	9	9.4	
**Histotype**					0.0558
Undifferentiated sarcoma	23	18.7	30	31.3	
Dedifferentiated liposarcoma	7	5.7	12	12.5	
Leiomyosarcoma	20	16.3	9	9.4	
Low grade fibromyxoid sarcoma/sclerosing epithelioid fibrosarcoma	10	8.1	4	4.2	
Myxofibrosarcoma	14	11.4	12	12.5	
Myxoid/round cell liposarcoma	21	17.1	7	7.3	
Synovial sarcoma	6	4.9	5	5.2	
Other **	22	17.9	17	17.7	
**Molecular class**					**0.0131**
Simple	49	39.8	23	24.0	
Complex	74	60.2	73	76.0	
**FNCLCC histological grade**					0.1295
1	30	24.4	13	13.5	
2	41	33.3	35	36.5	
3	52	42.3	48	50.0	
**Limitation**					0.1020
Well-circumscribed	63	51.2	41	42.7	
Focally infiltrative	45	36.6	33	34.4	
Diffusely infiltrative	15	12.2	22	22.9	
**Resection**					0.4564
R0	69	56.1	49	51.0	
R1	54	43.9	47	49.0	
**Type of surrounding tissue**					0.2313
Adipose	49	39.8	46	47.9	
Muscle	74	60.2	50	52.1	
**Adjuvant chemotherapy**					0.1646
No	105	85.4	75	78.1	
Yes	18	14.6	21	21.9	
**Adjuvant radiotherapy**					**0.0371**
No	20	16.3	29	30.2	
Second	93	75.6	58	60.4	
Third	10	8.1	9	9.4	
**Complete remission after treatment**					0.8736
No	11	8.9	8	8.3	
Yes	112	91.1	88	91.7	
**Metastasis**					0.8707
No	82	66.7	65	67.7	
Yes	41	33.3	31	32.3	
**Local recurrence**					**0.0021**
No	113	91.9	74	77.1	
Yes	10	8.1	22	22.9	
**Vital status**					NA
Alive	73	59.3	60	62.5	
Dead	50	40.7	36	37.5	
**Cause of death**					NA
Dead of other cause	10	20.0	9	25.0	
Dead of this cancer	38	76.0	22	61.1	
Unknown	2	4.0	5	13.9	
**First event to occur**					NA
Local relapse	8	6.5	16	16.7	
Metastasis	36	29.3	27	28.1	
Death	12	9.8	6	6.3	
No event	67	54.5	47	49.0	
**Lost of follow-up**					NA
No	121	98.4	96	100.0	
Yes	2	1.6	0	0.0	
**Total**	**123**	**100.0**	**96**	**100.0**	**219**

*: vascular, nervous or bone involvement. ** other: rhabdomyosarcoma, alveolar soft part sarcoma, angiosarcoma, clear cell sarcoma, epithelioid hemangioendothelioma, epithelioid sarcoma, extraskeletal myxoid chondrosarcoma, extraskeletal osteosarcoma, high risk solitary fibrous tumor, liposarcoma pleomorphic, malignant mesenchymoma, malignant peripheral nerve sheath tumor, malignant rhabdoid tumor, malignant tenosynovial giant cell tumor, undifferentiated sarcoma NOS. FNCLCC: Fédération Nationale des Centres de Lutte Contre le Cancer, R0: microscopically margin-negative resection, R1: microscopically margin-positive resection, fmTLS: fully mature tertiary lymphoid structures.

**Table 2:** Prognostic factors associated with 15-years overall survival (OS).

	Univariate Analysis				Multivariate Analysis			
Baseline Factors	Hazard Ratio (HR)	HR Lower Conf. Limit	HR Upper Conf. Limit	*p* Value (Chi-2)	Hazard Ratio (HR)	HR Lower Conf. Limit	HR Upper Conf. Limit	*p* Value (Chi-2)
**Sex**								
Female	1				-			
Male	0.95	0.62	1.45	0.811	-	-	-	-
**Age at diagnosis (years)**								
<65	1				1			
≥65	4.54	2.82	7.31	**<** **0.001**	4.03	2.31	7.02	**<** **0.001**
**Tumor site**				0.802				-
Lower limb	1				-			
Trunk wall	1.16	0.71	1.91	0.555	-	-	-	-
Upper limb	0.94	0.46	1.90	0.860	-	-	-	-
**Tumor depth**				0.423				-
Deep	1				-			
Superficial	0.67	0.27	1.66	0.384	-	-	-	-
Superficial and deep	1.25	0.75	2.08	0.396	-	-	-	-
**Tumor size**				**<** **0.001**				**<** **0.001**
<5 cm	1				1			
5 to 10 cm	1.84	0.77	4.40	0.168	1.83	0.74	4.50	0.189
>10 cm	4.75	2.03	11.13	<0.001	4.59	1.85	11.40	0.001
**Multifocality**								
No	1				-			
Yes	1.06	0.58	1.96	0.846	-	-	-	-
Locoregional extension *								
No	1				1			
Yes	2.03	1.14	3.60	**0.016**	1.96	1.01	3.80	**0.046**
**Histotype**				**0.042**				**0.006**
Undifferentiated sarcoma	1				1			
Dedifferentiated liposarcoma	1.02	0.46	2.27	0.957	0.44	0.18	1.03	0.060
Leiomyosarcoma	1.70	0.93	3.09	0.084	1.62	0.84	3.11	0.147
Low grade fibromyxoid sarcoma/Sclerosing epithelioid fibrosarcoma	***	***	***	***	***	***	***	***
Myxofibrosarcoma	0.80	0.39	1.68	0.561	0.65	0.30	1.38	0.263
Myxoid/round cell liposarcoma	0.18	0.05	0.59	0.005	0.34	0.10	1.19	0.092
Synovial sarcoma	0.98	0.38	2.57	0.969	3.14	1.09	9.10	0.035
Other **	1.04	0.55	1.94	0.911	1.67	0.85	3.28	0.140
**Molecular class**								
Simple	1				-			
Complex	3.21	1.78	5.79	**<** **0.001**	-	-	-	-
**FNCLCC histological grade**				**0.001**				-
1	1				-			
2	2.23	0.97	5.15	0.059	-	-	-	-
3	3.83	1.74	8.43	<0.001	-	-	-	-
**Limitation**				0.302				**0.018**
Well circumscribed	1				1			
Focally infiltrative	1.40	0.88	2.22	0.155	1.68	1.01	2.77	0.044
Diffusely infiltrative	0.97	0.52	1.83	0.933	0.64	0.31	1.30	0.214
**Resection**								
R0	1				1			
R1	2.70	1.73	4.24	**<** **0.001**	1.93	1.20	3.11	**0.007**
**Type of surrounding tissue**								
Adipose	1				-			
Muscle	1.15	0.74	1.78	0.548	-	-	-	-
**Adjuvant chemotherapy**								
No	1				-			
Yes	0.55	0.31	0.98	**0.041**	-	-	-	-
**Adjuvant radiotherapy**				0.068				-
No	1				-			
Second	1.06	0.61	1.84	0.826	-	-	-	-
Third	0.32	0.11	0.97	0.043	-	-	-	-
**Semi-quantitative TLS density score**								-
3+	1				-			
2+	1.39	0.79	2.45	0.247	-	-	-	-
1+	1.40	0.80	2.47	0.242	-	-	-	-
**TLS status**								
fmTLS^−^	1				-			
fmTLS^+^	0.85	0.55	1.31	0.466	-	-	-	-
**fmTLS^+^ and semi-quantitative density score 2+/3+**								
No	1				-			
Yes	0.84	0.54	1.30	0.426	-	-	-	-

*: vascular, nervous or bone involvement. ** other: rhabdomyosarcoma, alveolar soft part sarcoma, angiosarcoma, clear cell sarcoma, epithelioid hemangioendothelioma, epithelioid sarcoma, extraskeletal myxoid chondrosarcoma, extraskeletal osteosarcoma, high risk solitary fibrous tumor, liposarcoma pleomorphic, malignant mesenchymoma, malignant peripheral nerve sheath tumor, malignant rhabdoid tumor, malignant tenosynovial giant cell tumor, undifferentiated sarcoma NOS. ***: could not be computed due to the low sample size and the absence of deaths, FNCLCC: Fédération Nationale des Centres de Lutte Contre le Cancer, R0: microscopically margin-negative resection, R1: microscopically margin-positive resection, TLS: tertiary lymphoid structure, fmTLS: fully mature TLS.

**Table 3:** Prognostic factors associated with 15-year time to locoregional progression (TTLRP).

	Univariate Analysis				Multivariate Analysis			
Baseline Factors	Hazard Ratio (HR)	HR Lower Conf. Limit	HR Upper Conf. Limit	*p* Value (Chi-2)	Hazard Ratio (HR)	HR Lower Conf. Limit	HR Upper Conf. Limit	*p* Value (Chi-2)
**Sex**								
Female	1				-			
Male	1.71	0.82	3.54	0.152	-	-	-	-
**Age at diagnosis (years)**								
<65	1				1			
≥65	2.88	1.39	5.95	**0.004**	2.53	1.22	5.26	**0.013**
**Tumor site**				0.257				-
Lower limb	1				-			
Trunk wall	1.51	0.67	3.40	0.316	-	-	-	-
Upper limb	2.09	0.82	5.30	0.122	-	-	-	-
**Tumor depth**				0.326				-
Deep	1				-			
Superficial	2.10	0.78	5.64	0.141	-	-	-	-
Superficial and deep	1.31	0.55	3.12	0.542	-	-	-	-
**Tumor size**				0.257				-
<5 cm	1				-			
5 to 10 cm	0.77	0.31	1.87	0.557	-	-	-	-
>10 cm	0.76	0.28	2.04	0.582	-	-	-	-
**Multifocality**								
No	1				-			
Yes	1.71	0.70	4.15	0.240	-	-	-	-
Locoregional extension *								
No	1				-			
Yes	1.88	0.73	4.89	0.194	-	-	-	-
**Histotype**				0.211				-
Undifferentiated sarcoma	1				-			
Dedifferentiated liposarcoma	0.80	0.26	2.42	0.692	-	-	-	-
Leiomyosarcoma	0.14	0.02	1.06	0.057	-	-	-	-
Low grade fibromyxoid sarcoma/Sclerosing epithelioid fibrosarcoma	***	***	***	***	-	-	-	-
Myxofibrosarcoma	0.70	0.25	1.93	0.494	-	-	-	-
Myxoid/round cell liposarcoma	0.10	0.01	0.73	0.024	-	-	-	-
Synovial sarcoma	0.30	0.04	2.29	0.246	-	-	-	-
Other **	0.52	0.19	1.44	0.210	-	-	-	-
**Molecular class**								
Simple	1				-			
Complex	4.24	1.48	12.10	**0.007**	-	-	-	-
**FNCLCC histological grade**				**0.045**				-
1	1				-			
2	3.21	0.70	14.67	0.132	-	-	-	-
3	5.47	1.28	23.41	0.022	-	-	-	-
**Limitation**				0.078				-
Well circumscribed	1				-			
Focally infiltrative	2.33	1.03	5.26	0.042	-	-	-	-
Diffusely infiltrative	2.45	0.96	6.22	0.060	-	-	-	-
**Margin**								
R0	1				-			
R1	2.06	1.01	4.17	**0.046**	-	-	-	-
**Type of surrounding tissue**								
Adipose	1				-			
Muscle	0.87	0.43	1.75	0.688	-	-	-	-
**Adjuvant chemotherapy**								
No	1				-			
Yes	0.84	0.36	1.96	0.680	-	-	-	-
**Adjuvant radiotherapy**				**0.027**				-
No	1				-			
Second	0.40	0.19	0.85	0.017	-	-	-	-
Third	0.23	0.05	1.03	0.055	-	-	-	-
**Semi-quantitative TLS density score**				0.132				-
3+	1				-			
2+	0.35	0.12	0.96	0.042	-	-	-	-
1+	0.68	0.30	1.53	0.349	-	-	-	-
**TLS status**								
fmTLS^−^	1				-			
fmTLS^+^	2.69	1.27	5.68	**0.009**	-	-	-	-
**fmTLS^+^ and semi-quantitative density score 2+/3+**								
No	1				1			
Yes	3.01	1.45	6.24	**0.003**	2.68	1.28	5.59	**0.009**

*: vascular, nervous or bone involvement. ** other: rhabdomyosarcoma, alveolar soft part sarcoma, angiosarcoma, clear cell sarcoma, epithelioid hemangioendothelioma, epithelioid sarcoma, extraskeletal myxoid chondrosarcoma, extraskeletal osteosarcoma, high-risk solitary fibrous tumor, liposarcoma pleomorphic, malignant mesenchymoma, malignant peripheral nerve sheath tumor, malignant rhabdoid tumor, malignant tenosynovial giant cell tumor, undifferentiated sarcoma NOS. ***: could not be computed due to the low sample size, FNCLCC: Fédération Nationale des Centres de Lutte Contre le Cancer, R0: microscopically margin-negative resection, R1: microscopically margin-positive resection, TLS: tertiary lymphoid structure, fmTLS: fully mature TLS.

## Data Availability

The datasets generated and analyzed during the current study are not publicly available due to patient privacy restrictions and French regulatory constraints on medical data sharing. De-identified data may be made available from the corresponding author upon reasonable request and subject to approval by the appropriate institutional data protection office.

## Supplementary Materials

The following supporting information can be downloaded at: https://www.mdpi.com/article/10.3390/cancers18111685/s1, Figure S1: Pathological workflow for tertiary lymphoid structures (TLS) screening; Figure S2: Morphological screening for tertiary lymphoid structures (TLS) in sarcoma samples; Figure S3: Semi quantitative score of tertiary lymphoid structures (TLS); Table S1: Antibodies and immunohistochemical techniques; Table S2: Clinicopathological variables; Table S3. Semi-quantitative density score of TLS in TLS+ patients; Table S4: Overall characteristics of lymphoid aggregates in the cohort; Table S5: Comparison of lymphoid aggregates in patients with fmTLS^+^ versus fmTLS^−^ tumors; Table S6: Prognostic factors associated with 15-years time to distant progression (TTDP); Table S7: Immune status according to transcriptome versus TLS pathological status in the tran-scriptomic cohort (n = 126 patients); Table S8: Association of transcriptomic immune status and OS, TTDP and TTLRP in univariate analysis; Table S9: Contingency analysis of transcriptomic immune status and TLS status.

## Abbreviations

The following abbreviations are used in this manuscript:

CI	Confident Interval
FDC	Follicular Dendritic Cell
FNCLCC	Fédération Nationale des Centres de Lutte Contre le Cancer
ICI	Immune Checkpoint Inhibitor
IHC	Immunohistochemistry
MRI	Magnetic Resonance Imaging
OS	Overall Survival
TLS	Tertiary Lymphoid Structures
iTLS	Immature TLS
mTLS	mature TLS
fmTLS	fully Mature TLS
TTDP	Time to Distant Progression
TTLP	Time to Locoregional Progression
STS	Soft-Tissue Sarcoma
