# Supplementary material for: Beyond Surgical Margins: Fully Mature Tertiary Lymphoid Structures (fmTLSs) Are Predictive Biomarkers for Local Recurrence in Primary Soft-Tissue Sarcomas
Source: Cancers (Basel). 2026 May 22;18(11):1685. doi: 10.3390/cancers18111685 (PMC13255587; doi:10.3390/cancers18111685)
Supplement: Supplementary file 1 [file cancers-18-01685-s001.zip › cancers-4239141-supplementary/cancers-4239141-Supplementary figure.pdf]

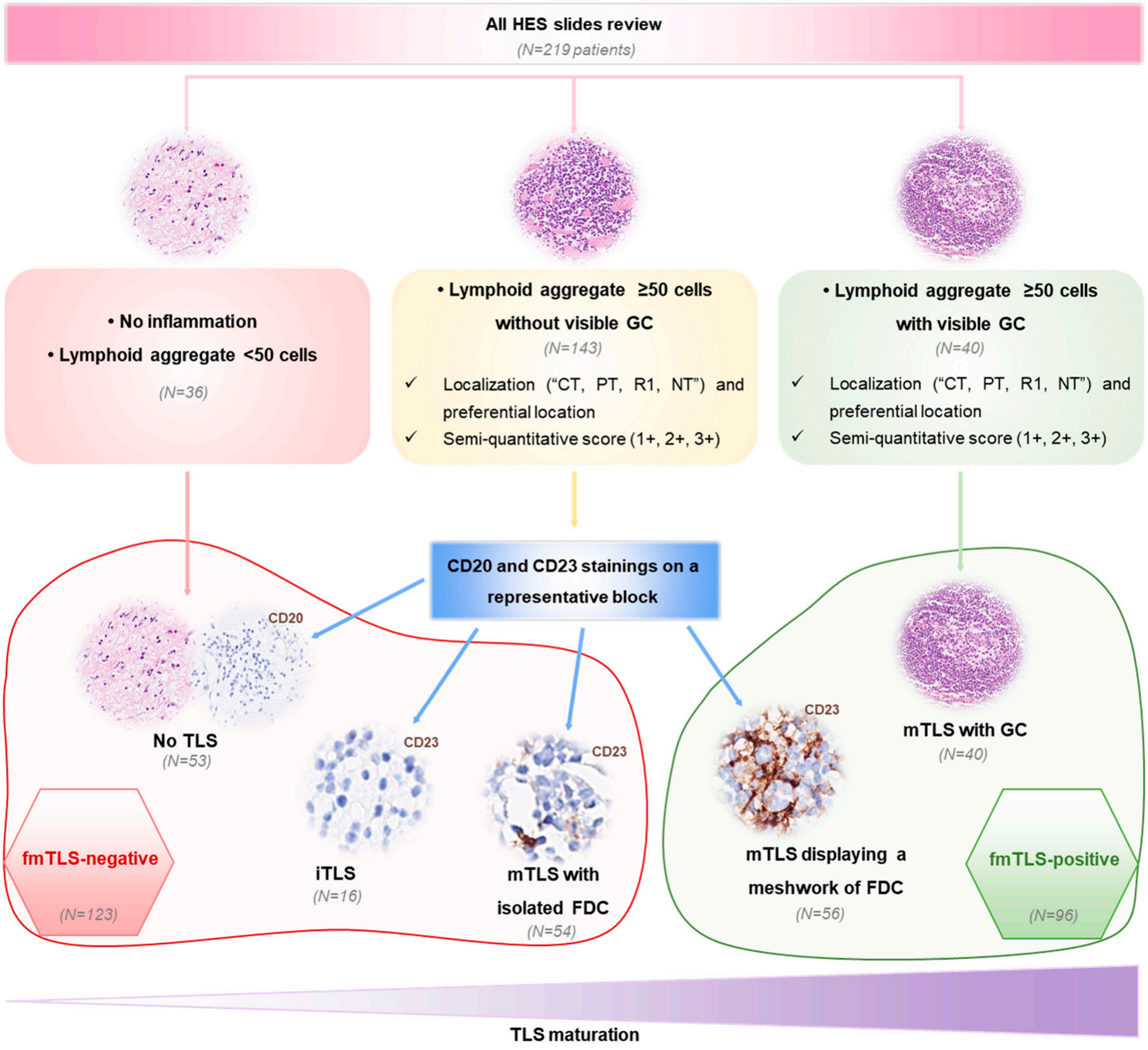

Supplementary Figure S1 :Pathological workflow for tertiary lymphoid structures (TLS) screening. The figure depicts the screening method used to classify the TLS status of each samples. HES: Hematoxylin-eosin-saffron; GC: germinal center; iTLS: immature TLS; mTLS: mature TLS; fmTLS: fully mature TLS; CT: TLS within the tumor bulk (intermingled with tumor cells); PT: TLS at periphery of the tumor; R1: TLS present beyond the tumor invasion front up to 1cm from the tumor; NT: TLS located in non-tumoral tissue beyond 1cm of the tumor.

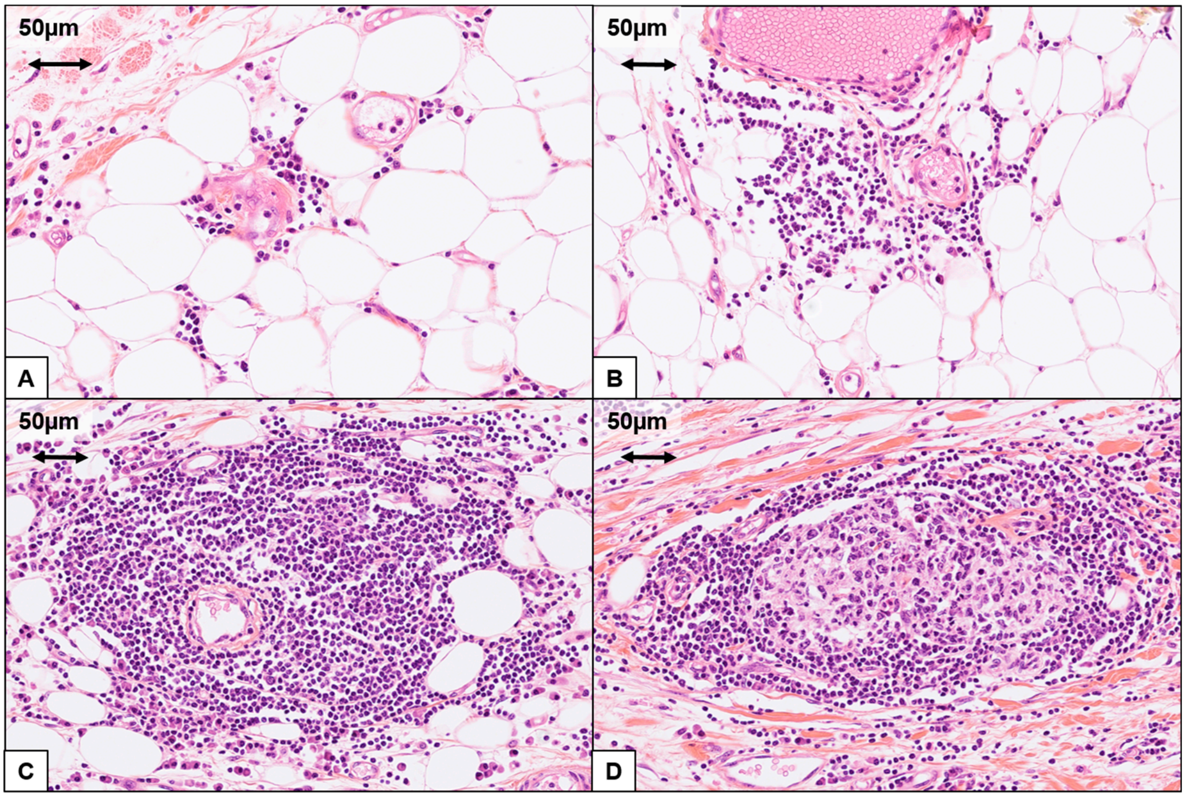

Supplementary Figure S2 :Morphological screening for tertiary lymphoid structures (TLS) in sarcoma samples. Lymphoid infiltrates were classified with the hematoxylin-eosin-saffron (HES) slides according to their appearance and size as shown in these representative examples of undifferentiated pleomorphic sarcoma (UPS): (A) absence of TLS detected on HES which do not yield to complementary techniques in the absence of significant infiltrate of lymphoid cells (<50 lymphocytes or scattered). (B) and (C) The detection of aggregated lymphoid cells with ≥50 cells yielded to complementary IHC (CD20 and CD23 immunostainings) to ascertain the B cell nature of the infiltrate and the maturity of the TLS. (D) Mature TLS (mTLS) could occasionally be diagnosed solely on HES when a germinal center is visible. All histology slides are stained with hematoxylin-eosin-saffron (HES). The scale bars indicate 50µm in size for all pictures.

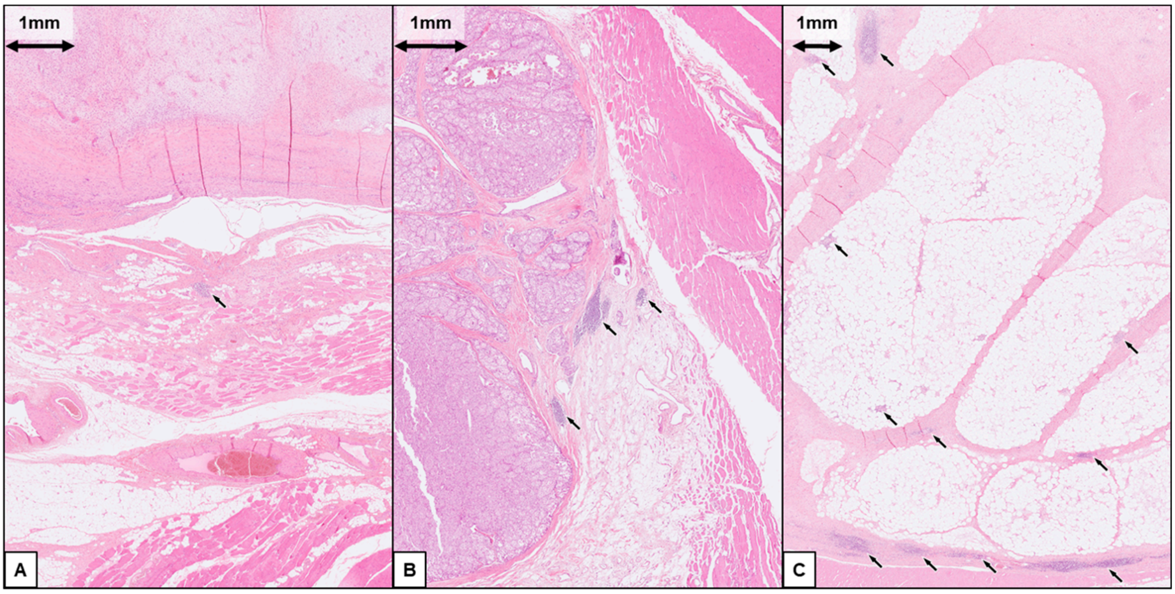

Supplementary Figure S3: Semi quantitative score of tertiary lymphoid structures (TLS). A semi-quantitative TLS' density score was based on the average number of lymphoid aggregates per slide, estimated on all slides analyzed. (A) Low grade fibromyxoid sarcoma showing 1+ score, with only 1 TLS spotted in this slide and no TLS detected in other slides (1+ score: less than 1 TLS per slide). (B) Alveolar soft part sarcoma displaying up to 3 TLS per slide, corresponding to 2+ score (2+ score: 1 to 5 TLS per slide). (C) Dedifferentiated liposarcoma with more than 5 TLS per slide, illustrating 3+ score (3+ score: more than 5 TLS per slide). All histology slides were stained with hematoxylin-eosin-saffron (HES). The scale bars indicate 1mm in size for all pictures. Arrows point to TLS.
